# Imaging cardiac ATTR amyloid

**DOI:** 10.1186/1750-1172-10-S1-I10

**Published:** 2015-11-02

**Authors:** Philip N Hawkins

**Affiliations:** 1 National Amyloidosis Centre, UCL, London, UK

## 

The heart is the principal site of involvement in non-hereditary ATTR amyloidosis and a major driver of treatment options and prognosis in hereditary forms of the disease. The goals of cardiac imaging in amyloidosis are to aid diagnosis, provide prognostic information, track disease progression and evaluate response to therapy.

Echocardiography has long been the mainstay of cardiac evaluation in amyloidosis, particularly that of diastolic dysfunction, but its precision is limited and both inter and intra-observer variability constrain its capacity to detect changes. The recent refinement of myocardial strain imaging provides more reproducible and more sensitive evaluation of systolic impairment, which may be overlooked in amyloidosis, and the longitudinal strain pattern of relative apical sparing strongly supports diagnosis of amyloidosis.

Two investigations that are transforming our understanding of cardiac amyloidosis are bone scintigraphy and cardiovascular magnetic resonance (CMR).

CMR with gadolinium contrast has proved to be invaluable for identification of cardiac amyloid, showing characteristic patterns of global subendocardial and transmural late gadolinium enhancement associated with abnormal myocardial and blood pool kinetics. The recent refinement of phase-sensitive inversion recovery sequence is highly sensitive and specific, producing findings that are virtually pathognomonic for amyloid. CMR is inferior to echocardiography for evaluating diastolic function but can assess the heart's structure and systolic function with greater accuracy and precision. A key advantage of CMR is its unique ability to give insight about tissue composition through myocardial tissue characterization. T1 mapping studies can quantify the massive expansion of the extracellular space caused by amyloid deposition as well as evaluating the myocyte response to it, i.e. associated hypertrophy or cell loss. The ability to independently track changes in amyloid load and myocardial cell mass will be invaluable in assessing new therapies. One significant limitation of CMR is its incompatibility with most implanted pacemakers and ICDs, an issue that may be addressed by the development of dynamic equilibrium CT, in which gated five minute contrast-enhanced scans have shown excellent potential for diagnosis and quantification of cardiac amyloid.

It has been known for decades that bone scintigraphy tracers can localize to cardiac amyloid deposits, but the enormous potential of this tool has only been fully realized in recent years. Studies have confirmed that ^99m^Tc-3, 3-diphosphono-1, 2-propanodicarboxylic acid (DPD) localizes to the heart in patients with cardiac ATTR amyloidosis with incredible sensitivity and high specificity. The mechanism remains unknown, but virtually all patients with clinically significant cardiac ATTR amyloidosis show Grade 2 or greater localization to the heart using the simple Perugini Grade 1-3 scoring system. Indeed, scans in apparently healthy individuals who have undergone predictive genetic testing for hereditary ATTR amyloidosis have shown that Grade 1 DPD uptake occurs before symptoms or abnormalities on echo or CMR have developed, reflecting the earliest evidence of cardiac ATTR amyloid. DPD scintigraphy is not completely specific for ATTR amyloid in that positive scans occur in a small proportion of patients with (usually very advanced) cardiac AL amyloidosis and some other very rare types, but Grade 2 or 3 cardiac uptake in the presence of a consistent echo and/or CMR and in the absence of a monoclonal gammopathy is in practice sufficient to make a non-invasive diagnosis of cardiac ATTR amyloidosis.

